# Socioeconomic variation of multimorbidity in Colombian older adults

**DOI:** 10.1038/s41598-021-02219-w

**Published:** 2021-11-23

**Authors:** Silvia Marcela Ballesteros, José Moreno-Montoya, Wilhelmus Johannes Andreas Grooten, Pedro Barrera-López, José A. De la Hoz-Valle

**Affiliations:** 1 Clinical Studies and Clinical Epidemiology Division, Fundación Santa Fe de Bogotá, Calle 119 A 7-49, Bogotá, Colombia; 2 Department of Neurobiology, Care Sciences and Society, Division of Physical Therapy, Karolinska Institutet, 141 83 Huddinge, Sweden; 3 Women’s Health and Allied Health Professionals Theme, Medical unit Occupational Therapy and Physiotherapy, Karolinska University Hospital, 171 77 Stockholm, Sweden

**Keywords:** Risk factors, Epidemiology

## Abstract

Multimorbidity (MM) prevalence among older adults is increasing worldwide. Variations regarding the socioeconomic characteristics of the individuals and their context have been described, mostly in high-income settings. However, further research is needed to understand the effect of the coexistence of infectious diseases along with socioeconomic factors regarding MM. This study aims to examine the variation of MM regarding infectious diseases mortality after adjusting for socioeconomic factors. A cross-sectional multilevel study with a nationally representative sample of 17,571 Colombian adults of 60 years of age or older was conducted. Individual socioeconomic, demographic, childhood and health related characteristics, as well as group level variables (multidimensional poverty index and infectious diseases mortality rate) were analyzed. A two-level stepwise structural equation model was used to simultaneously adjust for the individual and contextual effects. Multimorbidity prevalence was 62.3% (95% CI 61.7–62.9). In the multilevel adjusted models, age, female sex, having functional limitations, non-white ethnicity, high body mass index, higher income, physical inactivity and living in urban areas were associated with multimorbidity among the sample for this study. The median odds ratio for multidimensional poverty was 1.18 (1.16–1.19; p = 0.008) and for infectious diseases was 1.25 (1.22–1.28; p = 0.014). This paper demonstrates that MM varies regarding the mortality of infectious diseases and shows a strong association between MM and poverty in a low-middle income country. Differences in the factors involved in the etiology of multimorbidity are expected among wealthy and poor countries regarding availability and prioritization of health services.

## Introduction

Multimorbidity (MM), considered as the co-existence of two or more chronic conditions^1,2^ has been estimated to affect approximately 50 million people in Europe^3^ and one third of adults in Latin American countries^4^. In Colombia, among the registries of health services provided between 2012 and 2016, almost 6 million correspond to patients living with multimorbidity per year^5^. This figure is expected to increase since the prevalence of MM pursue advances along with the demographic transition^6^. Estimates in Europe report figures rising from 11% in adults aged 25–44 years to 65% for those aged 65–84 years, and up to 82% in those 85 years and older^7^. A similar trend is observed for Colombia with figures ranging from 33.1% in adults to 51.3% in people aged 60 or older^5^. Moreover, MM has been associated with poor health outcomes including functional decline^8–10^, excess of mortality^11,12^, decrease in quality of life levels^10^ and high economic costs of care due to increased hospitalization rates^13^ and polypharmacy^14^.

Variations in the prevalence of MM have also been associated with demographic and socioeconomic characteristics, being higher in women, single, widowed or divorced^15^, persons of lower socioeconomic levels and in those living in disadvantage settings^1,10,16^. Socioeconomic factors such as low-educational status^16,17^, childhood adversity^18^, and reduced lifetime earnings are related to an increased risk of developing MM in late life^19^. Likewise, previous research have indicated that MM rates are higher among population groups with greater difficulties accessing to drinking-water and energy for lighting sources^20^, as well as in areas with higher rates of unemployment and household overcrowding^21,22^. In particular, the prevalence of MM has been reported to be almost twice as high in areas of high socioeconomic deprivation (11.0%, 95% CI 10.9–11.2), compared to more affluent areas (5.9%, 5.8–6.0)^21^.

As it has been widely recognized, poor populations usually face the simultaneous effects of both chronic and infectious diseases. Similar to the association of chronic conditions with socioeconomic deprivation, those living in poorer communities are also more vulnerable to communicable diseases than the most affluent ones^23^, and these effects could be more serious in older adults due to the immunosenescence that make this population more susceptible to infections^24^. As long as the effect of the coexistence of infectious diseases along with socioeconomic factors regarding MM is not recognized, particular measures of prevention or even health needs could be omitted in regions where communicable diseases are endemic. Therefore, in this paper we examined the variation of MM regarding infectious diseases mortality after adjusting for the effects of socioeconomic factors.

## Methods

### Study design

A cross-sectional multilevel study was developed using secondary data from the Colombian population-based survey Health, Wellbeing and Aging (Salud, Bienestar y Envejecimiento—SABE), carried out in 2015. The SABE study included 23,694 individuals aged over 60 years living in urban and rural areas of the 32 Colombian departments (i.e., states). Participants were selected by a probabilistic, multistage and stratified sampling design. Data collection was completed using in-person surveys. For the analyses, a sample of 17,571 participants was used once records with missing information were excluded. Detailed information about the SABE study and the sampling method can be found elsewhere^25^.

### Variables

For the analysis “Multimorbidity” was considered the dependent variable, defined as the coexistence of two or more non-communicable diseases: diabetes, hypertension, cancer, lung disease (chronic obstructive pulmonary disease, asthma, bronchitis or emphysema), heart disease, stroke, joint diseases (arthritis, rheumatism or osteoarthritis), osteoporosis, depressive symptoms and other mental illnesses (mental or psychiatric problem). Except for depression, which was assessed with the Short Form of the Geriatric Depression Scale^26^, all morbidities were self-reported. A score of 6 or more was used as indicator of depression in the population^26,27^.

The independent variables of interest were both at individual and at group level. At individual level, the following categorical and continuous sociodemographic characteristics were used: Sex (female/male (reference category)), Age (continuous), Marital status (single, widowed or divorced/married or living with a partner (ref)), Ethnicity (non-white/white (ref)), Income (mean income less than $7.83/day/income of $7.83/day/more than $7.83/day (ref)), Educational level (secondary or lower level/higher level (ref)), Victim of armed displacement, defined as ever been displaced by armed conflict or violence (yes/no (ref)), Area of residence (rural/urban (ref)). Health and lifestyle-related variables included were Functional limitation defined as having a Barthel score of < 100^28^ (yes/no (ref)), Body Mass Index (BMI) (low (< 22)/overweight/obesity (> 27)/normal weight (22–27) (ref))^29^, Physical inactivity or a low level score of physical activity in the short-form International Physical Activity Questionnaire—IPAQ-SF^30^ (yes/no (ref)) and Smoking (current or former smoker/nonsmoker (ref)). Childhood-related factors were also included as Self-perceived childhood economic situation (fair/poor/good (ref)) and Self-perceived childhood health status (poor or fair/good (ref)).

At state-level, we used the prevalence of the multidimensionally poor^31^ for 2018^32^ and infectious diseases mortality rate for 2016^33^. To evaluate multidimensional poverty (MP), 5 dimensions with 15 indicators are measured, including: education, childhood and youth conditions (school attendance, childcare services), employment (informality, long-term unemployment), health (access, insurance), access to public utilities (water source, sewer system) and housing conditions (floors and walls material, overcrowding)^31^. Those deprived in 5 or more indicators are considered as multidimensionally poor^34^.

### Statistical analysis

Descriptive analysis of the individual characteristics was based on the absolute and relative frequencies with 95% confidence intervals (95% CI) for categorical variables, and measures of central tendency and dispersion (mean and standard deviation (SD)) for quantitative variables. To identify differences between baseline characteristics, independent X^2^ test and *t*-test analyses were developed for categorical and continuous variables respectively. Variables with p-values below 25% were considered for the adjusted models. A first one-level logistic model was used to evaluate the associations between the individual variables with MM, and significant variables (p < 0.05) were included in a multilevel stepwise backward model. To evaluate the variability of MM prevalence across states, the median OR (MOR)^35^ was calculated. Correlation was evaluated between MM and MP, between MM and infectious diseases mortality, and between infectious diseases mortality and MP, using Pearson correlation coefficients. A two-level structural equation model (SEM) was used to assess the association among individual and contextual variables with MM. The effect of state-level variables that were not measured, due to secondary data availability, was incorporated into the model through a latent variable. MP, BMI, physical activity and functionality were considered correlated in the model^36^. Sample weights were used in all the analyses.

### Ethics declarations

All methods in the present study were carried out in accordance with relevant guidelines and regulations. Ethics approval was granted by the Institutional Committee of Human Ethics of the Fundación Santa Fe de Bogotá Hospital. The approval ID is CCEI-11861-2020. As clinical data was used, participants provided written informed consent before enrolling in the SABE study^25^.

## Results

The overall prevalence of MM in the Colombian sample who were 60 years of age or older was 62.3% (95% CI 61.7–62.9%), the average age was 70.8 years (SD = 8.2) and 57.3% (56.7–58.0%) were women. Most frequent diseases in the population were depression, 56.9% (56.2–57.6), hypertension, 53.9% (53.2–54.6), and joint disease, 26.0% (25.3–26.6). Those living with two or more conditions suffered mainly of hypertension and depression (25.5%), hypertension and joint disease (16.3%), and hypertension and diabetes (12.5%) (Table 1).

**Table 1:** Population proportion in every comorbidities combination.

	Diabetes	Cancer	Lung disease	Heart disease	Stroke	Joint disease	Mental illness	Depression	Osteoporosis	Total (95% CI)
Hypertension	12.4	2.7	6.4	10.8	3.5	16.3	5.6	25.5	7.2	53.9 (53.2–54-6)
Diabetes		0.8	2.1	3.5	1.1	5.1	1.9	7.8	2.4	16.4 (15.9–17.0)
Cancer			0.8	0.9	0.3	1.5	0.5	2.2	0.8	4.4 (4.1–4.7)
Lung disease				2.8	0.7	3.8	1.5	5.0	2.1	10.2 (9.8–10.6)
Heart disease					1.5	4.7	2.1	6.4	2.5	13.6 (13.1–14.1)
Stroke						1.3	0.8	2.4	0.6	4.6 (4.3–4.9)
Joint disease							3.5	12.4	6.9	26.0 (25.3–26.6)
Mental illness								4.1	1.7	8.5 (8.1–8.9)
Depression									5.8	56.9 (56.2–57.6)
Osteoporosis										11.5 (11.1–12.0)

Crude comparisons at individual-level showed that all variables were significantly associated with MM except for educational status and smoking (Table 2). Once adjusted, the effects that remained significant were age, sex, having functional limitations, body mass index (BMI), income, physical inactivity, non-white ethnicity and living in urban areas (Table 3).

**Table 2:** Multimorbidity and individual characteristics.

Variable	Total (n = 17,571)		With Multimorbidity (n = 10,615)		Without Multimorbidity (n = 6956)		OR (CI 95%)	p
	N	%	N	%	N	%		
**Sex**								< 0.001
Female	9783	55.7	6752	63.6	3036	43.7	2.22 (2.10–2.34)	
Male	7783	44.3	3863	36.4	3920	56.3	Ref	
**Marital status**								< 0.001
Single. Widowed. Divorced	7597	43.3	4804	45.3	2793	40.2	1.23 (1.16–1.31)	
Other	9968	56.7	5807	54.7	4161	59.8	Ref	
**Ethnicity**								< 0.001
Non-white	12,492	71.1	7307	68.8	5185	74.5	0.75 (0.71–0.81)	
White	5079	28.9	3308	31.2	1771	25.5	Ref	
**Income**^**a**^								0.001
Less than $7.83/day	9620	64.8	5669	63.9	3951	66.4	0.90 (0.93–0.98)	
$7.83/day	2588	17.5	1562	17.6	1026	17.2	0.94 (0.85–1.05)	
More than $7.83/day	2623	17.7	1647	18.6	976	16.4	Ref	
**Education level**								0.437
Secondary or lower	16,187	92.3	9763	92.2	6424	92.5	0.96 (0.85–1.07)	
Upper than secondary	1352	7.7	830	7.8	522	7.5	Ref	
**Physical inactivity**								< 0.001
Yes (Low IPAQ-SF score)	7390	42.1	5081	47.9	2309	33.2	2.01 (1.91–2.13)	
No (Moderate to High IPAQ-SF score)	10,170	57.9	5526	52.1	4644	66.8	Ref	
**Victim of armed displacement**								< 0.001
Yes	3220	18.3	1850	17.4	1370	19.7	0.86 (0.80–0.93)	
No	14,349	81.7	8765	82.6	5584	80.3	Ref	
**Perceived childhood economic situation**								< 0.001
Poor	2901	16.6	1877	17.8	1024	14.8	1.34 (1.23–1.47)	
Fair	7406	42.3	4535	43.0	2871	41.4	1.16 (1.08–1.24)	
Good	7182	41.1	4145	39.3	3037	43.8	Ref	
**Functional limitation**								< 0.001
Yes (Barthel score < 100)	2791	15.9	2341	22.0	450	6.5	3.76 (3.48–4.07)	
No (Barthel score of 100)	14,780	84.1	8274	78.0	6506	93.5	Ref	
**Smoking**								0.807
Former or current smoker	9094	51.8	5502	51.8	3592	51.7	1.01 (0.95–1.07)	
Nonsmoker	8473	48.2	5111	48.2	3362	48.3	Ref	
**Area of residence**								< 0.001
Rural	4493	25.6	2383	22.5	2110	30.3	0.67 (0.63–0.71)	
Urban	13,078	74.4	8232	77.5	4846	69.7	Ref	
**Body Mass Index**								< 0.001
Low BMI (˂22 kg/m^2^)	2097	15.3	1016	12.4	1081	19.6	0.76 (0.70–0.83)	
High BMI (˃27 kg/m^2^)	6155	45.0	4152	50.9	2003	36.3	1.65 (1.54–1.77)	
Normal BMI (22—27 kg/m^2^)	5432	39.7	2994	36.7	2438	44.1	Ref	
**Self-perceived childhood health status**								< 0.001
Poor or fair	1836	10.5	1208	11.4	628	9.0	1.30 (1.17–1.43)	
Good	15,700	89.5	9382	88.6	6318	91.0	Ref	
Age (Mean (SD))	69.2 (7.2)		70.0 (7.3)		68.1 (6.8)		–	< 0.001

*BMI* Body Mass Index.

^a^Dollar values for year 2015.

**Table 3:** Adjusted associations between individual variables and MM using a multiple logistic stepwise model.

	Adj. OR	95% CI	p-value
Age (years)	1.04	1.03–1.06	< 0.001
Female sex	2.27	1.84–2.81	< 0.001
Functional limitation	3.29	2.28–4.74	< 0.001
Physical inactivity	1.38	1.13–1.68	0.002
Income of less than $7.83 per day^a^	0.72	0.55–0.95	0.020
Income of $7.83 per day^a^	0.93	0.68–1.27	0.651
Rural residence	0.58	0.44–0.77	< 0.001
Non-white ethnicity	0.73	0.58–0.92	0.008
Low BMI (˂ 22 kg/m^2^)	0.73	0.55–0.99	0.040
High BMI (˃ 27 kg/m^2^)	1.31	1.04–1.64	0.021

*BMI* Body Mass Index.

^a^Dollar values for year 2015.

At state-level, the prevalence of MM varied between 42.6% (36.6–48.9) and 74.1% (69.2–78.5), where the ages ranged from 60 to 93 years (mean = 69.0; SD = 7.7) and from 60 to 96 years (mean = 69.3; SD = 7.5), respectively. MP varied from 4.4 to 65.0 (mean = 28.2; SD = 15.1), where ages ranged from 60 to 101 years (mean = 70.8; SD = 8.2) and from 60 to 90 years (mean = 68.7; SD = 6.3), respectively. Infectious diseases mortality rate varied from 12.6/100,000 to 51.0/100,000 (mean = 35.9/100,000; SD = 1.7) (Table 4), and the ages ranged from 60 to 96 years (mean = 69.3; SD = 7.5) and from 60 to 90 years (mean = 68.7; SD = 6.3). Correlation between MM prevalence and MP was of − 0.80 (p < 0.001), between MM and infectious diseases mortality of − 0.40 (p = 0.022), and between infectious diseases mortality and MP of 0.18 (p = 0.320).

**Table 4:** Multimorbidity prevalence by state and state-level variables.

State	Multimorbidity prevalence	Multidimensional poverty	Infectious diseases mortality rate
San Andrés	74.1	8.9	12.6
Putumayo	71.6	25.1	27.1
Risaralda	71.1	12.5	44.2
Bogotá D.C	71.1	4.4	23.8
Quindío	71.1	16.2	46.2
Antioquia	69.7	17.1	35.5
Cundinamarca	69.3	11.5	27.6
Valle del Cauca	68.8	13.6	39.0
Boyacá	66.5	16.6	23.0
Santander	65.9	12.9	37.5
Caldas	65.1	15.3	29.4
Norte de Santander	62.6	31.5	46.1
Cauca	62.3	28.7	21.6
Meta	62.2	15.6	44.8
Guaviare	61.7	33.5	26.4
Nariño	61.0	33.5	16.5
Atlántico	60.3	20.1	50.6
Bolívar	59.3	32.4	37.4
Amazonas	58.8	34.9	39.2
Chocó	58.5	45.1	36.2
Tolima	57.8	23.5	38.8
La Guajira	56.7	51.4	30.9
Arauca	55.7	31.8	42.6
Casanare	55.4	19.1	50.0
Caquetá	54.6	28.7	29.5
Córdoba	54.0	36.7	38.2
Sucre	53.8	39.7	41.1
Vichada	53.7	55.0	37.5
Huila	52.3	19.2	39.1
Magdalena	51.8	38.6	43.3
Cesar	50.9	33.2	47.8
Guainía	44.1	65.0	51.0
Vaupés	42.6	59.4	31.6

**Figure 1:**
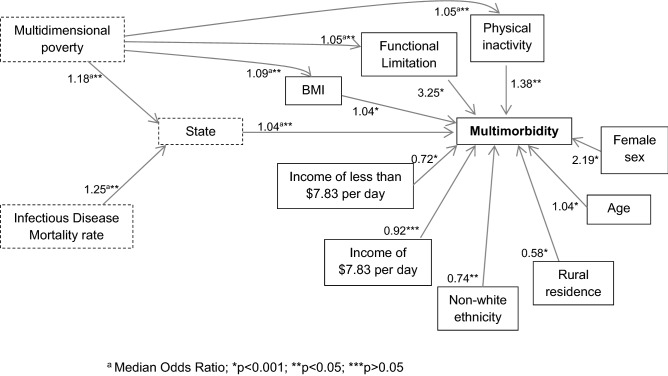
Final structural equation model (SEM). Odds ratio, median odds ratio and P-values. ^a^Median odds ratio; *p < 0.001; **p < 0.05; ***p > 0.05.

Adjusted analyses indicated a significant inter-state variability. The MOR for MP was 1.18 (1.16–1.19; p = 0.008) and for infectious diseases was 1.25 (1.22–1.28; p = 0.014). The association between MP and MM was also mediated by physical inactivity (p = 0.001), BMI (p = 0.002) and functional limitation (p = 0.001). All individual variables were directly associated with MM in the model (Fig. 1).

## Discussion

This study found a 63% prevalence of multimorbidity among Colombian older adults when considering a list of ten chronic conditions and a cut-off point of 2 or more concurrent morbidities for its definition, including diabetes, hypertension, cancer, lung disease, heart disease, stroke, joint disease, osteoporosis, depressive symptoms and other mental illnesses. Compared to findings from other authors, this corresponds to earlier studies in developing countries such as Brazil and Argentina, in which the prevalence of MM among older adults has been reported to be of 66.3%^37^ and 60.6%^38^, respectively. In this study, around 30% of those living with MM had poor functional status, which reinforces the need for preventive, coordinated and non-fragmented care^21^.

In this study MM prevalence increases with age, is higher in women and in individuals with high body mass index as reported before^6,19^. Our results also showed that in the states with higher infectious diseases mortality rate, MM was less likely to be reported. In deprived contexts, where infectious diseases are more prevalent, decision makers are compelled to assign significant portions of resources to the attention and diagnosis of these diseases displacing the care of chronic conditions, in particular MM^39^.

In contrast to previous studies our results indicate lower levels of MM in people living in middle/high income or less deprived settings. Barnett et al.^21^ and Salisbury et al.^22^, in a Scottish and English population, respectively, found that people living in more deprived settings were more likely to be living with MM. These dissimilarities could derive from methodological matters including the use of clinical records and a more specific list of morbidities for the definition of MM in these studies^21,22^. Also, a better opportunity to be diagnosed or have a medical evaluation in more affluent settings could partly explain them; in fact, in less prosperous contexts, limited availability of resources is associated with a lower rate in diagnosis of non-communicable diseases^40–42^.

Furthermore, the use of self-assessments instead of using medical records, could have led to a difficulty to accurately recall diagnosed chronic diseases. In addition, ongoing treatment might be higher in more affluent populations with better access to medical care which can improve the diagnosis self-reports^40^.

Living in a rural residence, being non-white ethnicity and low household income, all of which have been related with deprivation, were also associated with lower risk of reporting multiple chronic conditions. Similar findings have been found by previous research^40,43^, and are also related with the accessibility to public and private services. Therefore, our results might reflect an under-reported prevalence of MM in poor regions and populations due to difficulties in the access to health services and limited resource availability for adequate diagnosis.

Our findings contrast mainly with studies conducted in high-income countries. However, comparisons among national-level figures must consider differences among access to healthcare, services coverages and deprivation level among the poor, which might not be the same between countries. Moreover, an under-diagnosis or the lag in diagnosis also affects prevalence estimations.

Limitations in our study need to be considered. The self-reported measures of conditions (except depressive symptoms) can underestimate the prevalence; also, effects could be underestimated due to differences between analyzed and excluded individuals, who were significantly older, had a higher proportion of women and had less education and income. Likewise, as secondary data were used, care needs or the severity of the diseases could not be included for the analyses. Unequal weights regarding the type and severity of the conditions are warranted to assess the impact of multimorbidity in the population^40^.

Considering the above, this paper demonstrates that MM varies between areas regarding mortality of infectious diseases and shows a strong association between MM and poverty in a low-middle income setting. This study found lower levels of MM among individuals in states of high infectious disease mortality rate and in less deprived settings. Further research is needed to better understand the role of deprivation due the scarce number of publications coming from the developing world. Considerable differences in the factors involved in the etiology of MM are expected among high-, middle- and low-income countries regarding availability and prioritization of health services.

## Data Availability

The data that support the findings of this study are available from the Colombian Ministry of Health and Social Protection but restrictions apply to the availability of these data, which were used under license for the current study, and so are not publicly available. Data are however available from the authors upon reasonable request and with permission of the Ministry of Health and Social Protection. The datasets regarding group level variables generated during and/or analysed during the current study are available in the National Administrative Department of Statistics repository, https://www.dane.gov.co/index.php/estadisticas-por-tema/pobreza-y-condiciones-de-vida/pobreza-y-desigualdad/pobreza-monetaria-y-multidimensional-en-colombia-2018#pobreza-por-departamentos-2018.
